# CRISPR-Cas9 screening reveals a distinct class of MHC-I binders with precise HLA-peptide recognition

**DOI:** 10.1016/j.isci.2024.110120

**Published:** 2024-05-27

**Authors:** Tom A.W. Schoufour, Anneloes van der Plas - van Duijn, Ian Derksen, Marije Melgers, Jacqueline M.F. van Veenendaal, Claire Lensen, Mirjam H.M. Heemskerk, Jacques Neefjes, Ruud H.M. Wijdeven, Ferenc A. Scheeren

**Affiliations:** 1 Department of Cell and Chemical Biology, Oncode Institute, Leiden University Medical Center, 2333 ZA Leiden, Zuid-Holland, the Netherlands; 2 Department of Medical Oncology, Leiden University Medical Center, 2333 ZA Leiden, Zuid-Holland, the Netherlands; 3 Department of Dermatology, Leiden University Medical Center, 2333 ZA Leiden, Zuid-Holland, the Netherlands; 4 Department of Hematology, Leiden University Medical Center, 2333 ZA Leiden, Zuid-Holland, the Netherlands; 5 Department of Functional Genomics, Center for Neurogenomics and Cognitive Research (CNCR), Vrije Universiteit Medical Center, 1007 MB Amsterdam, Noord-Holland, the Netherlands

**Keywords:** Biochemistry, Bioinformatics, Biological sciences, Immunology, Natural sciences

## Abstract

Human leukocyte antigen (HLA) class-I molecules present fragments of the cellular proteome to the T cell receptor (TCR) of cytotoxic T cells to control infectious diseases and cancer. The large number of combinations of HLA class-I allotypes and peptides allows for highly specific and dedicated low-affinity interactions to a diverse array of TCRs and natural killer (NK) cell receptors. Whether the divergent HLA class-I peptide complex is exclusive for interactions with these proteins is unknown. Using genome-wide CRISPR-Cas9 activation and knockout screens, we identified peptide-specific HLA-C∗07 combinations that can interact with the surface molecules CD55 and heparan sulfate. These interactions closely resemble the HLA class-I interaction with the TCR regarding both the affinity range and the specificity of the peptide and HLA allele. These findings indicate that various proteins can specifically bind HLA class-I peptide complexes due to their polymorphic nature, which suggests there are more interactions like the ones we describe here.

## Highlights

• CRISPR-Cas9 screens can be used to identify receptor-ligand interactions
• HLA class-I tetramers can bind surface molecules allotype and peptide specifically
• Tetramerized HLA class-I can be used to study low-affinity interactions

## Graphical abstract

**Figure:**
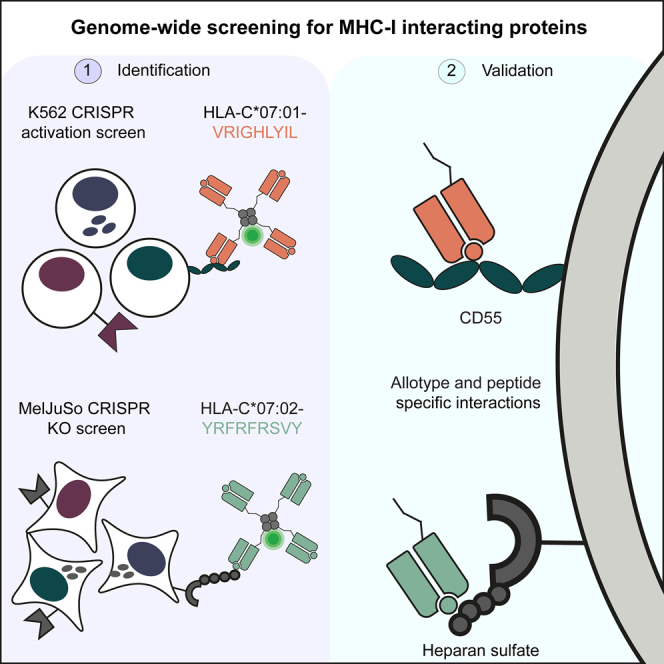

## Introduction

Major histocompatibility complex (MHC) class-I molecules are fundamental to the adaptive immune system as they present intracellular peptides to the T cell receptor (TCR) on cytotoxic CD8^+^ T cells. Proper binding of MHC class-I complexes to TCRs is dependent on the combination of the human leukocyte antigen (HLA) allotype (*HLA-A*, *HLA-B*, and *HLA-C* in humans), its corresponding allele, and the presented peptide sequence.^1^^,^^2^ Peptides presented by HLA class-I are generally 8 to 11 amino acids long and contain a few anchor residues for docking in the peptide-binding groove of the HLA class-I molecule, while the other residues are exposed for T cell recognition.^3^^,^^4^^,^^5^ The presence of various HLA class-I allotypes with different docking sites ensures that a high variety of intracellular peptides can be presented.^6^^,^^7^^,^^8^ This ensures specific and sensitive T cell recognition, creating a system to detect intracellular antigens.^9^^,^^10^

The TCR contains a hypervariable region which is the result of gene rearrangements and additional mutations. The process of positive and negative selection ensures that these TCRs recognize HLA class-I molecules in the context of an associated peptide but with a relatively low affinity.^11^^,^^12^ HLA class-I molecules can modulate immune responses by binding to various receptors on immune cells, including several receptors beyond the TCR.^13^^,^^14^^,^^15^ These receptors include members of the leukocyte immunoglobulin-like receptor (LILR) family and killer immunoglobulin-like receptors (KIRs), which prevent myeloid cells and natural killer (NK) cells from activation, respectively.^16^^,^^17^ KIRs are predominantly expressed on NK cells and display allotype-specific binding to HLA class-I, with HLA-C allotypes as their primary targets.^18^^,^^19^ Moreover, KIRs exhibit variable peptide sequence recognition, resulting in differential binding for various percentages of peptide sequences.^20^ LILRB1 and LILRB2 on the other hand are expressed mainly on myeloid cells, including macrophages, and bind to most HLA class-I allotypes in a peptide sequence-independent manner.^21^^,^^22^ HLA class-I interactions with LILRs and KIR family members are part of an essential surveillance mechanism to eliminate cells that have lost HLA class-I expression.^23^^,^^24^ While these interactions are well characterized, it is unknown whether there are more proteins that interact with HLA class-I molecules. Moreover, efforts are underway to develop and evaluate specific antibodies targeting defined peptides in complex with a particular HLA allele for potential therapeutic applications.^25^^,^^26^

In order to identify possible additional interactors for recombinant HLA class-I, we performed genome-wide CRISPR-Cas9-based activation and knockout (KO) screens, using recombinant HLA class-I tetramers as baits. These screens identified CD55 and heparan sulfate chains as binders to recombinant HLA class-I. We characterized these low-affinity interactions in detail, revealing they are HLA class-I allele and peptide specific. The characterization of these interactions presents an undefined class of interactions that HLA class-I peptide complexes can engage in.

## Results

### Genome-wide CRISPR-Cas9 activation screen reveals CD55 as an interactor of HLA-C∗07:01-VRIG

**Figure 1:**
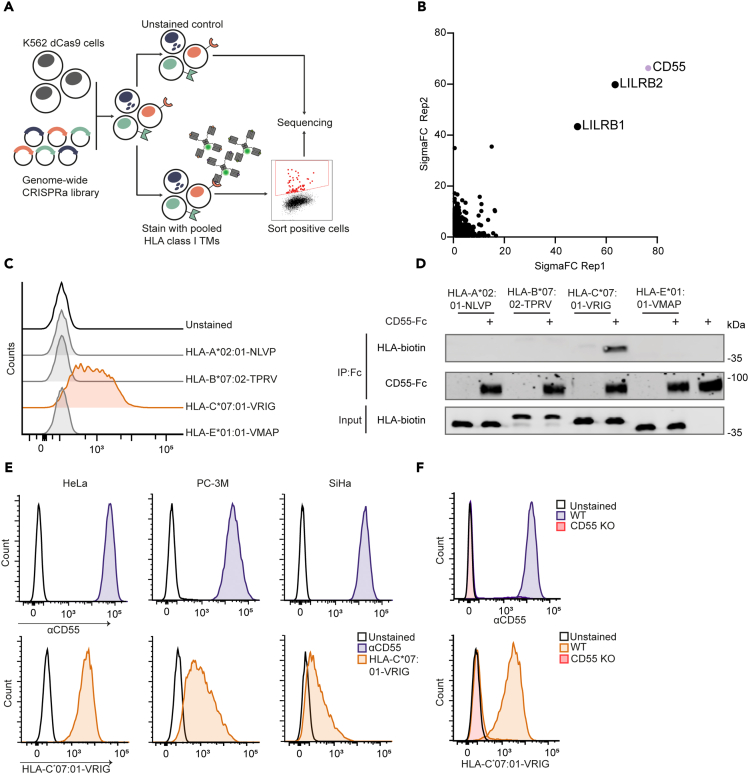
Receptor-ligand CRISPR-Cas9 activation screen reveals that CD55 interacts with HLA-C∗07:01-VRIG tetramers (A) Schematic of the receptor ligand CRISPR-Cas9 activation screen. K562 cells transduced with a genome-wide activation library were stained with a pool of three HLA tetramers (HLA-A∗02:01-NLVP, HLA-B∗07:02-TPRV, and HLA-C∗07:01-VRIG), and enriched gRNAs in stained cells were identified using NGS. (B) SigmaFC scores of genes from two replicate screens. SigmaFC scores were calculated using PinAplPy, and top hits are annotated. (C) K562 cells stably expressing dCas9 and transduced with a gRNA upregulating CD55 or a control guide were stained with the HLA-A, -B, -C, or tetramers as in (A) or with HLA-E∗01:01-VMAP tetramers and analyzed by flow cytometry. (D) *In vitro* co-immunoprecipitation of recombinant CD55-Fc with HLA-A∗02:01-NLVP, HLA-B∗07:02-TPRV, HLA-C∗07:01-VRIG, or HLA-E∗01:01-VMAP tetramers. (E) Three different cell lines (HeLa, PC-3M, or SiHa) that express CD55 endogenously were stained for CD55 (top) or with HLA-C∗07:01-VRIG tetramers (bottom) and analyzed by flow cytometry. (F) HeLa wild-type or HeLa CD55 KO cells were stained with αCD55 or HLA-C∗07:01-VRIG tetramers and analyzed by flow cytometry. All data except (B) represent at least three independent experiments. CRISPRa, CRISPR activation screen; TMs, tetramers; WT, wild-type; KO, knockout. Related to Figure S1 and Table S1.

To investigate whether there are other surface molecules that interact with HLA class-I in a systematic manner, we used recombinant HLA class-I tetramers as a bait in a genome-wide activation screen. For this, K562 cells were transduced with a genome-wide CRISPR-Cas9 activation library to create a pool of cells in which every cell overexpresses a different gene of the genome, including the cell surface proteins. Next, we stained these cells with a pool of three different randomly selected HLA-I tetramers, with a high-affinity peptide, one for each of the major HLA-alleles (A, B, and C). These tetramers included CMV-derived viral peptides NLVPMVATV (NLVP) and TPRVTGGGAM (TPRV) on HLA-A∗02:01 and HLA-B∗07:02, respectively, as well as a tumor MAGE-12 antigen-derived peptide VRIGHLYIL (VRIG) on HLA-C∗07:01. Cells that stained positive for HLA tetramers were isolated, and enriched gRNAs were detected using next-generation sequencing (NGS) (Figure 1A). Both replicates of these screens identified *LILRB1* and *LILRB2* as top hits, validating the screening. We detected similar enrichments for *CD55*, a glycosylphosphatidylinositol-anchored surface protein (Figures 1B; S1A; Table S1). Adequate sgRNA coverage was checked by calculating the Gini coefficient (Figure S1B). To validate the screening results and to determine which of the recombinant tetramers binds CD55, we generated K562 cells overexpressing CD55 (Figure S1C) and stained these with the individual tetramers. K562 cells overexpressing CD55 interacted exclusively with HLA-C∗07:01-VRIG tetramers, while the other tetramers and CMV-derived peptide VMAPRTLIL (VMAP) associated with HLA-E∗01:01 showed no binding (Figure 1C). To verify whether CD55 directly interacts with HLA-C∗07:01-VRIG, we performed *in vitro* co-immunoprecipitation experiments with recombinant Fc-tagged CD55 and all tetramers used in the screen and HLA-E∗01:01-VMAP. CD55 bound to the HLA-C∗07:01-VRIG tetramer contrary to HLA-A∗02:01-NLVP, HLA-B∗07:02-TPRV, or HLA-E∗01:01-VMAP (Figure 1D). Next, we verified that cancer cells with high CD55 expression, such as HeLa, PC-3M, and SiHa cells (Figure 1E, upper panel), were able to bind HLA-C∗07:01-VRIG tetramers (Figure 1E, lower panel). We established CD55 KO HeLa cells (Figure 1F, upper panel) as an additional control for the specificity of the HLA-C∗07:01-VRIG tetramer for CD55. HeLa CD55 KO cells did not interact with the HLA-C∗07:01-VRIG tetramer as shown by flow cytometry (Figure 1F, lower panel) and microscopy (Figure S1D). The binding of the HLA-C∗07:01-VRIG tetramer could be rescued by transient transfection of CD55 KO cells with a CD55 overexpression plasmid (Figure S1E), validating the specificity for CD55. Thus, our activation screen identified CD55 as an interactor of HLA-C∗07:01-VRIG.

### The interaction between CD55 and HLA-C∗07:01-VRIG is peptide and allotype specific

**Figure 2:**
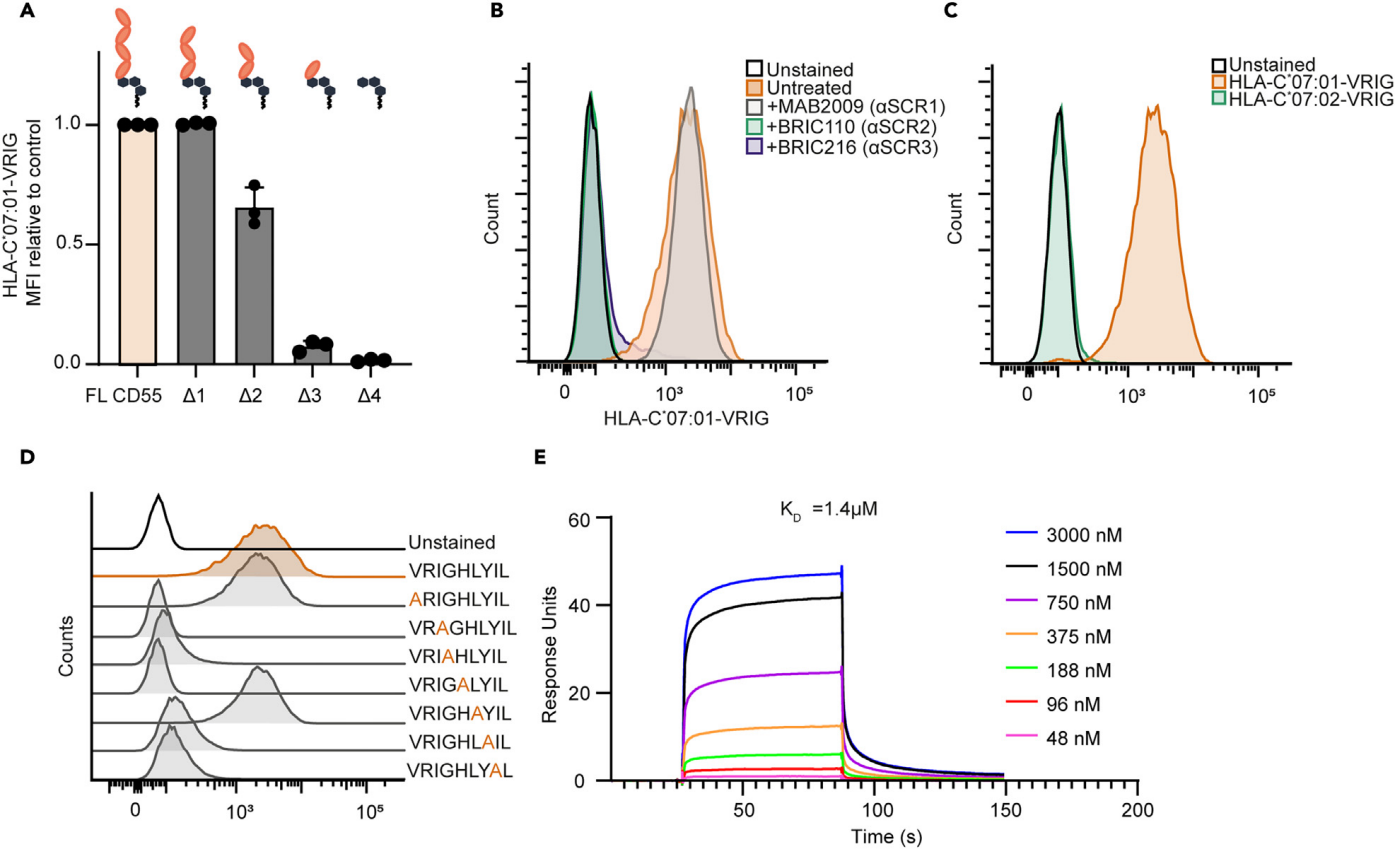
Interaction of CD55 with HLA-C∗07:01-VRIG tetramers is allotype and peptide specific (A) HEK293T cells were transfected with a plasmid containing GFP and a truncation mutant of CD55 and analyzed by flow cytometry. GFP+ positive cells were analyzed for staining with HLA-C∗07:01-VRIG. Each mutant removes an additional SCR domain from CD55. Data are represented as mean ± SD. (B) HeLa cells were stained with HLA-C∗07:01-VRIG tetramers after pre-incubation with CD55 blocking antibodies targeting different SCR domains on CD55 and analyzed by flow cytometry. (C) HeLa cells were stained with either HLA-C∗07:01 or HLA-C∗07:02 tetramers loaded with the VRIG peptide and analyzed by flow cytometry. (D) HeLa cells were stained with HLA-C∗07:01 tetramers loaded with different alanine mutants of the VRIGHLYIL peptide and analyzed by flow cytometry. (E) CD55-Fc was immobilized on a Prot-G chip for SPR data using HLA-C∗07:01-VRIG tetramers as analyte to determine interaction on and off rates and K_D_. Response units were measured with increasing concentrations of HLA-C∗07:01-VRIG tetramers. All data represent at least three independent experiments, except (E), which represents a biological duplicate. FL, full length. Related to Figure S2, Tables S2 and S3.

The extracellular region of CD55 consists of four sushi consensus repeat (SCR) domains and functions to inhibit the complement system or to bind CD97 to promote adhesion.^27^^,^^28^^,^^29^ To map the interaction with HLA-C∗07:01-VRIG, we generated CD55 truncation mutants that shorten CD55 by one SCR domain each time (Δ1- Δ4) and fused them to an IRES-GFP sequence to identify transfected cells (Figure S2A; Table S2). While cells expressing full length or Δ1 CD55 bound HLA-C∗07:01-VRIG, this interaction was reduced by about 50% upon elimination of SCR2 and was completely abrogated by additional removal of SCR3 (Figure 2A). Similarly, a blocking MAB2009 CD55 antibody targeting SCR1 did not affect the CD55 interaction with HLA-C∗07:01-VRIG, while antibodies targeting SCR2 and SCR3 (BRIC110 and BRIC216, respectively) almost fully blocked HLA-C∗07:01-VRIG tetramer staining of HeLa and PC-3M cells (Figure 2B; S2B), suggesting that HLA-C∗07:01-VRIG tetramers bind to a region at the interface of SCR2/3. Of note, all CD55-blocking antibodies were able to bind to HeLa wild-type but not CD55 KO cells (Figure S2C). Next, we decided to investigate which determinants of the HLA molecule are involved in the interaction with CD55. HLA-C allotype dependency was evaluated by employing HLA-C∗07:02 tetramers loaded with the identical VRIG peptide as the HLA-C∗07:01 variant. These allotypes exhibit only a minor amino acid variation, and both demonstrate a strong affinity for the VRIG peptide (Table S3). Interestingly, despite this slight difference, only the HLA-C∗07:01-VRIG tetramers exhibited binding to HeLa cells, in contrast to the HLA-C∗07:02 variants (Figure 2C). Both tetramers were properly folded as illustrated by their binding to LILRB1-expressing cells (Figure S2D). To delve deeper into peptide specificity, we conducted an alanine scan mutagenesis on the presented peptide. Single-site alanine substitutions were made on all residues of the VRIGHLYIL peptide, except the two anchor residues at position 2 and 9 of the peptide since these sites are essential for peptide binding to HLA-C∗07 (Table S3). Peptides were loaded on HLA-C∗07:01, and five out of seven mutations completely abolished interaction with HeLa cells (Figures 2D; S2E). Only the alanine modification of valine (V) at position 1 and leucine (L) at position 6 did not affect binding, suggesting that the other five amino acids are crucial for this interaction. None of the HLA-C∗07:01-VRIG alanine mutants were able to bind to HeLa CD55 KO cells, indicative of CD55-specific binding (Figure S2F).

MHC class-I has a low-affinity interaction with the TCR, which is why tetramers are used to increase avidity and to allow detection of the interaction. To determine the affinity of CD55 with HLA-C∗07:01-VRIG, we used SPR. Recombinant CD55-Fc was immobilized on a protein-G chip, and HLA-C∗07:01-VRIG tetramers or monomers were used as analyte. HLA-C∗07:01-VRIG tetramers interacted with CD55-Fc with a K_D_ of 1.4 μM (Figure 2E), while HLA-C∗07:01-VRIG monomers showed no detectable binding at the highest concentration (12 μM) (Figure S2G). Together, the interaction of HLA-C∗07:01-VRIG tetramers with CD55 resembles the MHC interaction with the TCR as highly specific and of low affinity for the HLA allele in combination with peptide.

### CRISPR-Cas9 activation and KO screens identify an additional HLA class-I binding partner

**Figure 3:**
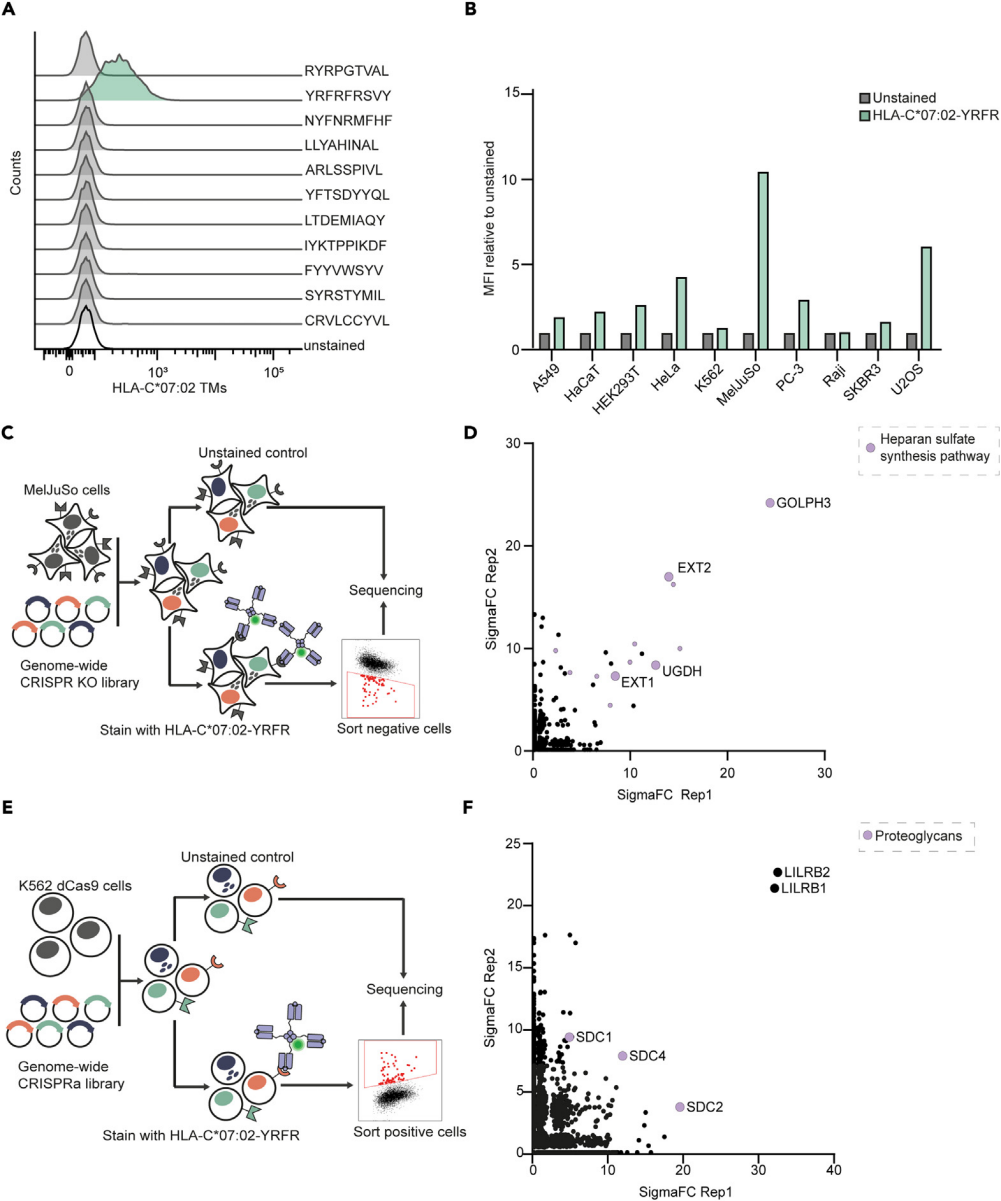
CRISPR-Cas9 activation and KO screens identify an interaction between HLA-C∗07:02-YRFR and heparan sulfate chains (A) HeLa cells were stained with a variety of HLA-C∗07:02 tetramers loaded with different peptides and analyzed by flow cytometry. (B) The indicated cell lines were stained with HLA-C∗07:02-YRFR tetramers and analyzed by flow cytometry. Staining is normalized to unstained levels of that cell line. (C) Schematic of CRISPR-Cas9 KO screen in MelJuSo cells. Cells were stained with HLA-C∗07:02-YRFR, and enriched gRNAs in non-binding cells were identified using NGS. (D) SigmaFC scores of genes from two replicate screens. SigmaFC scores were calculated using PinAplPy, and genes involved in the heparan sulfate biosynthesis pathway are depicted in purple. Hits used for further validation are annotated. (E) Schematic of the CRISPR-Cas9 activation screen. K562 cells stably expressing dCas9 transduced with a genome-wide activation library were stained with HLA-C YRFR∗07:02 tetramers, and enriched gRNAs in positive cells were identified. (F) SigmaFC scores of genes from two replicate screens. SigmaFC scores were calculated using PinAplPy, and top hits are annotated. Proteoglycans of interest are depicted in purple. CRISPRa, Crispr activation screen. Related to Figure S3, Table S1 and S4.

The HLA-C∗07:01-VRIG peptide complex could be considered a protein surface that creates a unique and distinct protein binding site. Consequently, it is plausible that other combinations of HLA and peptides may facilitate interactions with various proteins. To expand on this concept, we examined 116 recombinant HLA class-I tetramers, including our positive control HLA-C∗07:01-VRIG, with different alleles of HLA-A, -B, and -C, paired with a diverse range of peptides with varied physicochemical properties, to assess their binding to HeLa cells (Table S4). In one experiment with different peptides in HLA-C∗07:02, we found that one of the HLA-C∗07:02 tetramers stained HeLa cells, namely the HLA-C∗07:02-YRFRFRSVY (YRFR) TM (Figure 3A). All other 114 tetramers showed no staining to HeLa cells. Next, we stained 10 different cell lines with the HLA-C∗07:02-YRFR tetramer (Figure 3B), revealing broad expression of the HLA-C∗07:02-YRFR-target. Following this, we proceeded to conduct a genome-wide CRISPR-Cas9 KO screen in MelJuSo cells to uncover the binding partner interacting with HLA-C∗07:02-YRFR. Cells that stained negative for this tetramer were sorted out, and the isolated gDNA was analyzed by NGS (Figure 3C). Both replicates of the screen identified multiple genes involved in the heparan sulfate synthesis pathway (Figures 3D; S3A; Table S1), though the sgRNA coverage in this screen was relatively inequal (Figure S3B). We have identified multiple hits in both phases of the heparan sulfate biosynthesis pathway. Enzymes involved in the heparan sulfate biosynthesis comprise two main stages: first the polarization and elongation and second the synthesis of precursor molecules needed for heparan sulfate biosynthesis. Specifically, glycosyltransferases EXT1 and EXT2 are responsible for transferring N-acetyl glucosamine and glucuronic acid residues during the polarization and elongation step.^30^ Additionally, UGDH and GOLPH contribute to the heparan sulfate biosynthesis pathway. UGDH catalyzes the formation of a necessary precursor called UDP-alpha-D-glucuronate, while GOLPH aids in the trafficking of potentially the EXT1/2 enzymes through the Golgi apparatus.^31^^,^^32^ In parallel, we performed a genome-wide activation screen with HLA-C∗07:02-YRFR tetramers on K562 cells. Cells with increased binding were sorted out and sequenced (Figure 3E). Besides the expected *LILRB1* and *LILRB2*, this screen identified *SDC1*, *SDC2*, and *SDC4* as interactors with the HLA-C∗07:02-YRFR tetramer (Figures 3F; S3C; Table S1). Calculation of sgRNA coverage was done using the coefficient (Figure S3D). Syndecans (SDCs) are a family of heparan sulfate proteoglycan proteins,^33^ suggesting that our CRISPR KO and overexpression screens identify heparan sulfate chains as the molecule recognized by HLA-C∗07:02-YRFR.

### Heparan sulfate chains interact with the HLA-C∗07:02-YRFR HLA class-I allotype-peptide combination

**Figure 4:**
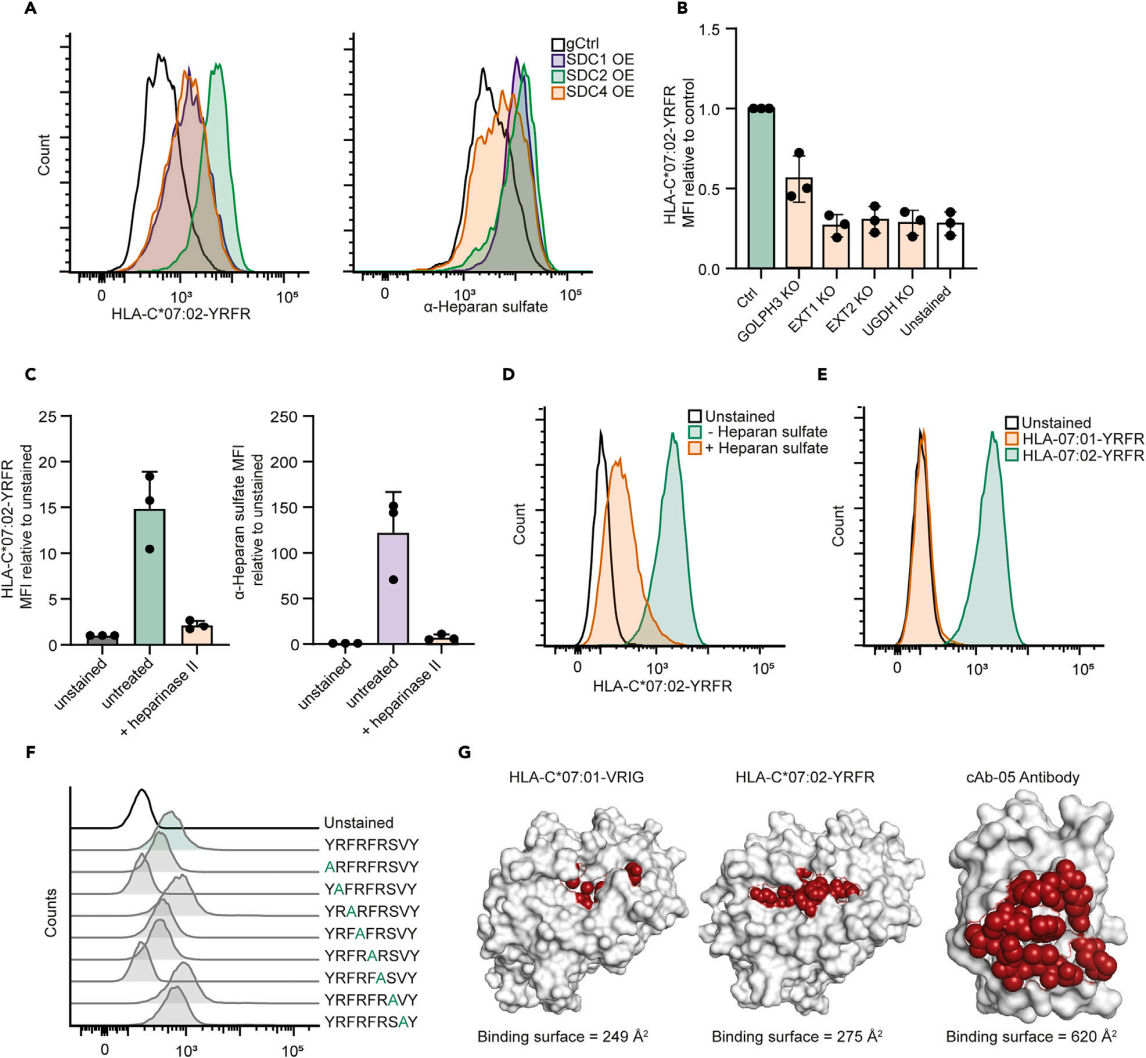
Interaction between HLA-C∗07:02-YRFR and heparan sulfate chain is allotype and peptide specific (A) K562 cells stably expressing dCas9 were transduced with a gRNA activating *SDC1*, *SDC2*, or *SDC4*, or a control guide and were stained with either HLA-C∗07:02-YRFR tetramers or an antibody targeting heparan sulfate chains prior to analysis using flow cytometry. (B) MelJuSo cells were transduced with a gRNA knocking out *GOLPH3*, *EXT1*, *EXT2*, or *UGDH*, stained with HLA-C∗07:02 tetramers, and analyzed by flow cytometry. Data are represented as MFI relative to the C∗07:02-YRFR tetramer staining on WT MelJuso cells. (C) MelJuSo cells were treated with heparinase II and subsequently stained with either HLA-C∗07:02-YRFR tetramers (left) or with an antibody targeting heparan sulfate chains (right), followed by analysis by flow cytometry. Data are relative staining to unstained levels. (D) MelJuSo cells were stained with HLA-C∗07:02-YRFR tetramers pre-incubated with recombinant heparan sulfate chains when indicated and analyzed by flow cytometry. (E) MelJuSo were stained with either HLA-C∗07:01 or HLA-C∗07:02 tetramers loaded with YRFR peptide and analyzed by flow cytometry. (F) MelJuSo cells were stained with HLA-C∗07:02 tetramers loaded with iterative alanine mutants of the YRFRFRSVY peptide and analyzed by flow cytometry. (G) Space-filling model of HLA-C∗07:01-VRIG, HLA-C∗07:02-YRFR, and the camelid antibody cAb-005. Shown is a top view of the different proteins. Residues identified to contact the binding partner of each molecule are depicted in red, and the calculated surface binding area is noted below the figure. Figures created using PyMol. All data represent at least three independent experiments, and bar graphs represent mean ± SD. gCtrl, guide control; SDC, syndecan; OE, overexpression; KO, knockout. Related to Figure S4 and Table S3.

To confirm the hits from both screens, we cloned the gRNAs that were most enriched in the screens into either the pXPR_502 (activation) or LentiCRISPRv2 (KO) vector.^26^^,^^27^ K562 cells transduced with guides overexpressing SDC1, -2, and -4 (Figures S4A and S4B) all showed increased levels of HLA-C∗07:02-YRFR staining (Figure 4A, left) as well as an increase in surface heparan sulfate levels determined using a heparan sulfate-specific antibody (Figure 4A, right). In parallel, we confirmed that multiple members of the heparan sulfate synthesis pathway are essential for HLA-C∗07:02-YRFR binding on MelJuSo cells. KO of heparan sulfate pathway genes *EXT1*, *EXT2*, or *UGDH* in MelJuSo cells, functionally validated by a loss of heparan sulfate at the cell surface (Figure S4C), all prevented binding of the HLA-C∗07:02-YRFR tetramer, while KO of *GOLPH3* reduced staining levels to around half of control cells (Figure 4B). Rescue of EXT1/2 expression by transient transfection restored HLA-C∗07:02-YRFR binding levels to wild-type levels (Figure S4D). As an additional control, heparan sulfate chains were enzymatically removed from the cell surface using Heparinase II, which abrogated HLA-C∗07:02-YRFR binding on MelJuSo (Figure 4C). Removal of the heparan sulfate chains was determined using a heparan sulfate chain-specific antibody. Since heparan sulfate chains act as a template for the binding of multiple proteins,^34^ we pre-incubated HLA-C∗07:02-YRFR with free heparan sulfate chains to test whether these could prevent HLA-C∗07:02-YRFR binding to the cells. Indeed, pre-incubation of HLA-C∗07:02-YRFR tetramers with free heparan sulfate chains almost completely prevented tetramer binding to MelJuSo (Figure 4D) and HeLa cells (Figure S4E), indicating that HLA-C∗07:02-YRFR interacts directly with the heparan sulfate chains. As a control, the interaction between HLA-C∗07:01-VRIG tetramers and CD55 was unaffected by free heparan sulfate (Figure S4F). To test for allele specificity, HLA-C∗07:01 tetramers loaded with the YRFR peptide were generated (Table S3). Only the HLA-C∗07:02-YRFR tetramers were able to bind to MelJuSo (Figure 4E) and HeLa cells (Figure S4G). Both tetramers were properly folded as they still bound LILRB1-expressing cells (Figure S4H). Next, we performed an alanine scan with the YRFR peptide on MelJuSo to determine which amino acids of the peptide are essential for the interaction with heparan sulfate chains (Table S3). Heparan sulfate is negatively charged and can bind proteins with positive charges from lysine (K) and arginine (R) residues,^28^ which are present on three positions in the YRFR peptide. The arginine residues at position 2 and 6 were essential for the interaction with heparan sulfate, while the alanine substitutions at positions 1, 4, and 5 only slightly decreased the interaction (Figures 4F; S4I). Interestingly, substitutions at position 3, 7, and 8 seemed to increase tetramer binding. Of note, pre-incubation with recombinant heparan sulfate reduced the binding of all alanine mutants to MelJuSo cells (Figure S4J). Based on the alanine scan and the allotype specificity of both HLA-C tetramer interactions, we modeled the HLA-C∗07:01-VRIG and HLA-C∗07:02-YRFR complexes with the amino acids that are potentially essential for their interactions (Figure 4G). Calculation of the potential binding surfaces of their interacting amino acids gives 249 Å^2^ and 275 Å^2^, respectively, which are relatively small compared to the binding surface that antibodies utilize. The single-domain camel antibody cAb-05 has a predicted binding surface of 620 Å^2^ with an affinity in the nanomolar range.^35^ This difference in antigen contact area could explain the difference in affinity between the HLA class-I-peptide combinations compared to antibodies. The affinity is more in line of that of TCRs for MHC molecules^11^^,^^12^ illustrating why these will only be detected by HLA tetramers unlike single HLA molecules. Together, these data present a second example of a surface molecule that specifically interacts with an HLA class-I peptide complex.

## Discussion

The divergent nature of the HLA class-I allotypes and the presented peptides have been analyzed extensively in the context of the interactions with the TCR, KIRs, and LILRBs.^20^^,^^36^ However, there is no reason to hypothesize that no other proteins have an affinity for defined HLA class-I allele-peptide combinations. Here we show that recombinant peptide HLA class-I complexes can indeed bind alternative molecules and present the technology on how to find these molecules. Through genome-wide CRISPR activation and KO screenings and binding assessment of 116 different tetramers, including different self- and viral peptide presenting HLA-A, -B, -C, and -E tetramers, two specific peptide-allotype combinations that bind entirely different ligands were identified: HLA-C∗07:01-VRIG to CD55 and HLA-C∗07:02 to heparan sulfate. These interactions are highly peptide and allotype specific, in a manner that resembles the canonical peptide HLA class-I TCR interactions.

The number of peptide-HLA allotype combinations is enormous, and each combination can potentially generate a different protein-binding surface, analogous to the way antibodies vary their protein-binding surface with their highly variable complementarity determining regions 1-3. Therefore, the antigenic space of peptide HLA class-I complexes should be large, which translates to a large potential to bind many different antigens with complementary surfaces. Compared to affinity-maturated antibodies, the affinity of these peptide HLA class-I complexes is lower and more comparable to the affinity of TCRs for MHC molecules. While maturated antibody-antigen interactions usually hold affinities in the nano- to picomolar range,^37^^,^^38^ the estimated affinity for the HLA-C∗07:01-VRIG interaction with CD55 was considerably lower, in the micromolar range. Although we observe a low-affinity interaction, we observe a high antigen specificity based on the CRISPR screens, comparable to TCRs. The high specificity of the HLA class-I complexes suggests they could be used as an alternative to antibodies, certainly when the avidity is increased using HLA class-I peptide tetramers. The combination of a low affinity and high-specificity interaction is potentially interesting in the context of agonistic reagents. Systematic examination of agonistic antibodies revealed that low-affinity antibody variants can induce more potent signaling and mediate increased receptor clustering.^39^ Intermediate affinity allows for repeated interactions with the same receptor, thereby inducing consecutive signaling, while simultaneously enhancing receptor clustering by binding two receptors simultaneously and continually repeating this process.^40^ MHC tetramers are excellent tools for this concept. Chemical synthesis and loading of peptides into the HLA class-I molecule allow introduction of chemical groups not present in nature and would allow further modifications of the HLA class-I binding surface for optimized affinities or other applications. By using temperature-dependent peptide exchange technology, many HLA class-I peptide complex combinations can be easily generated and tested for altered affinity and specificity.^41^^,^^42^^,^^43^ This process bears a resemblance to the affinity maturation observed in antibodies but is now under controlled laboratory conditions.

To identify HLA class-I-based binding partners, two strategies can be employed. First, to detect binding partners in a systematic manner, a pool of defined HLA class-I tetramers can be used as bait in a genome-wide activation screen as performed here. Secondly, when binding partners for a specific target are desired, the target antigen can be produced and fluorescently multimerized, whereby it serves as a bait to stain libraries of HLA class-I-based yeast display libraries.^44^^,^^45^ The HLA class-I-based yeast display libraries are currently used to find the peptide for specific TCRs; however, it would be interesting to use non-TCR targets.

HLA class-I tetramers were designed to specifically stain antigen-specific T cells and since then have been used in thousands of studies.^46^^,^^47^ Several of these studies have observed unexplainable background staining when using HLA allotype-peptide combinations to detect immune cells.^48^^,^^49^ This background staining only occurs for specific HLA tetramers on certain cell populations. It seems likely that tetramers with such background staining are binding unintended ligands in a similar manner as described here. Such specific HLA class-I tetramers could then be used in the genome-wide CRISPR screening setup described here to identify more ligand- and allotype-specific HLA binders. While our work enabled the swift identification of targets for two HLA-C class-I complexes, it also has some limitations. It remains uncertain whether these interactions occur under physiological conditions and whether they have any functional significance.

In summary, we provide a detailed characterization of two recombinant HLA-C peptide complexes and their binding to two different targets. We found these targets through unbiased genome-wide CRISPR-Cas9 activation and KO screens. The shared similarities regarding peptide and allotype specificity between the two HLA class-I complexes and their respective targets imply that these types of interactions could be more common. Exploring the protein-binding space of HLA class-I complexes may yield new interactions and potential applications in the activation or inactivation of surface receptors. The fact that the peptide can be easily modified allows for expansion of the chemical space limited in the case of antibodies.

### Limitations of the study

In this study, we have investigated interactions between surface molecules and tetramerized recombinant HLA class-I proteins. Although these findings imply there could be more interactions between specific HLA class-I complexes and other surface molecules, the sheer amount of possible combinations make them challenging to find. Additionally, since our interactions were characterized by tetramerized recombinant HLA class-I complexes, we do not know whether these interactions would occur with cell surface-bound HLA class-I.

## STAR★Methods

### Key resources table

**Table:** 

REAGENT or RESOURCE	SOURCE	IDENTIFIER
**Antibodies**		

Anti-Heparan Sulfate (clone F58-10E4)	AMSBIO	Cat: 370255-1; RRID: AB_10891554
Anti-human CD55 FITC	BioLegend	Cat: 311306; RRID: AB_314863
Anti-human CD55 (clone BRIC110)	ARP American Research Products	Cat: 08-9402-2; RRID: AB_1540745
Anti-human CD55 (clone BRIC216)	Bio-Rad	Cat: MCA914T; RRID: AB_1102203
Anti-human CD55 (clone 278803)	R&D Systems	Cat: MAB2009; RRID: AB_2075961
Anti-human Syndecan-2 APC (clone 305515)	R&D Systems	Cat: FAB2965A; RRID: AB_2182872
Anti-human Syndecan-4 APC (clone 336304)	R&D Systems	Cat: FAB29181A; RRID: AB_10888658
Anti-human CD55 APC	BioLegend	Cat: 311311; RRID: AB_2075856
Goat anti-mouse IgG APC	BioLegend	Cat: 405308; RRID: AB_315011
IRDye 680RD Goat anti-human IgG	LI-COR Biosciences	Cat: 926-68078; RRID: AB_10956144

**Bacterial and virus strains**		

5-alpha E. Coli	NEB	Cat: C2987I

**Chemicals, peptides, and recombinant proteins**		

Blasticidin	Thermo Fisher	Cat: A1113903
Bovine Serum Albumin	Sigma-Aldrich	Cat: A3912
Bromophenol blue	Merck	Cat: 108122
CD55-Fc	Sino Biological	Cat: 10101-H02H
DAPI	ThermoFisher	Cat: D1306
Glycerol	Sigma-Aldrich	Cat: 49770
Heparinase II	R&D Systems	Cat: 6336-GH-010
Heparan sulfate	SelleckChem	Cat: S5992
Lipofectamine 3000	Thermo Fisher	Cat: L3000008
MgCl2	Sigma-Aldrich	Cat: M1028
Milk powder	Sigma-Aldrich	Cat: M7409
NaCl	VWR	Cat: 27810
NP40	Merck Millipore	Cat: 492016
Paraformaldehyde, 4%	ThermoFisher	Cat: J61899.AK
Polybrene	Merck Millipore	Cat: TR-1003-G
Polyethyleneimine	Polyscience Inc.	Cat: 23966-1
ProLong® Gold Antifade Reagent	Cell Signaling Technology	Cat: 9071S
Protease inhibitors	Roche	Cat: 5056489001
Puromycin	Thermo Fisher	Cat: J672368EQ
Streptavidin IRDye 800CW	LI-COR	Cat: 926-32230
SuperBlock Blocking Buffer	Thermo Fisher	Cat: 37515
Tris-HCl	Combi Blocks	Cat: OR-5119
Tween 20	Sigma-Aldrich	Cat: P1379
β-mercaptoethanol	Millipore-Sigma	Cat: 63689
VRIGHLYIL	In-house	N/A
ARIGHLYIL	In-house	N/A
VRAGHLYIL	In-house	N/A
VRIAHLYIL	In-house	N/A
VRIGALYIL	In-house	N/A
VRIGHAYIL	In-house	N/A
VRIGHLAIL	In-house	N/A
VRIGHLYAL	In-house	N/A
YRFRFRSVY	In-house	N/A
ARFRFRSVY	In-house	N/A
YAFRFRSVY	In-house	N/A
YRARFRSVY	In-house	N/A
YRFAFRSVY	In-house	N/A
YRFRARSVY	In-house	N/A
YRFRFASVY	In-house	N/A
YRFRFRAVY	In-house	N/A
YRFRFRSAY	In-house	N/A
EVDPIGHVY	In-house	N/A
YVGKEHMFY	In-house	N/A
LTEGHSGNYY	In-house	N/A
LTQDLVQEKYLEY	In-house	N/A
YTAVVPLVY	In-house	N/A
NLVPMVATV	In-house	N/A
FTWEGLYNV	In-house	N/A
FVYGEPREL	In-house	N/A
KMVELVHFL	In-house	N/A
SLLMWITQC	In-house	N/A
SLLQHLIGL	In-house	N/A
NLVPMVATV	In-house	N/A
VLHDDLLEA	In-house	N/A
AQFPNHSFK	In-house	N/A
ATLPPFMRNK	In-house	N/A
RIIVPLNNR	In-house	N/A
SLFRAVITK	In-house	N/A
SVMGVYVGK	In-house	N/A
ATLPPFMCNK	In-house	N/A
ELFSYLIEK	In-house	N/A
ISDPTSPLRTR	In-house	N/A
LANDLMLIK	In-house	N/A
RIIVPLNNR	In-house	N/A
CYTAVVPLV	In-house	N/A
GYLQGLVSF	In-house	N/A
RWPSCQKKF	In-house	N/A
VAELVHFLL	In-house	N/A
YYCSVGYGF	In-house	N/A
TPRVTGGGAM	In-house	N/A
LPHNHTDL	In-house	N/A
APAPTAVVL	In-house	N/A
MPFATPMEA	In-house	N/A
RVRIAYPSL	In-house	N/A
TPMEAELARRSL	In-house	N/A
YPSLREAAL	In-house	N/A
ALKLKVAEL	In-house	N/A
ISKERAEAL	In-house	N/A
KPCAQAATL	In-house	N/A
TLKIRAEVL	In-house	N/A
GQAGFFPSPF	In-house	N/A
GQNLKYGEF	In-house	N/A
GQVRLRALY	In-house	N/A
NQLQVQHTY	In-house	N/A
RVIKNDSNY	In-house	N/A
APAPTAVVL	In-house	N/A
EVDPIGHVY	In-house	N/A
LPHQPLATY	In-house	N/A
LPRELFPPL	In-house	N/A
MPMQDIKMIL	In-house	N/A
CEDVPSGKL	In-house	N/A
GETKMVETAL	In-house	N/A
MEQLEQLEL	In-house	N/A
RESEEESVSL	In-house	N/A
YEDIHGTLHL	In-house	N/A
EEKRGSLHVW	In-house	N/A
NEDGSITNF	In-house	N/A
VEITPYKPTW	In-house	N/A
LAAAHPAAL	In-house	N/A
RPQHPGDAL	In-house	N/A
AAFDGRHSQTL	In-house	N/A
FAFGEPREL	In-house	N/A
FVYGEPREL	In-house	N/A
LAMPFATPM	In-house	N/A
SAYGEPRKL	In-house	N/A
VSMNPYQEL	In-house	N/A
LAAAHPAAL	In-house	N/A
RPQHPGDAL	In-house	N/A
AAFDGRHSQTL	In-house	N/A
FAFGEPREL	In-house	N/A
FVYGEPREL	In-house	N/A
SAYGEPRKL	In-house	N/A
VSMNPYQEL	In-house	N/A
VRIGHLYIL	In-house	N/A
AANPHSFVF	In-house	N/A
ARPTAPFTL	In-house	N/A
AYSDVAKRL	In-house	N/A
FFKELIQEF	In-house	N/A
FRPEHVSRL	In-house	N/A
FSNPYSIEY	In-house	N/A
LRPSTSRSL	In-house	N/A
MAPERVASL	In-house	N/A
NRPKALNAL	In-house	N/A
NYFGAQPHY	In-house	N/A
YRFRFRSVY	In-house	N/A
ARGPESRLL	In-house	N/A
LRTRFVYHL	In-house	N/A
VRFFFPSL	In-house	N/A
AANPHSFVF	In-house	N/A
ARPTAPFTL	In-house	N/A
AYSDVAKRL	In-house	N/A
FFKELIQEF	In-house	N/A
FRPEHVSRL	In-house	N/A
FSNPYSIEY	In-house	N/A
LRPSTSRSL	In-house	N/A
MAPERVASL	In-house	N/A
NRPKALNAL	In-house	N/A
NYFGAQPHY	In-house	N/A
VRIGHLYIL	In-house	N/A
ARGPESRLL	In-house	N/A
HPAALHQRL	In-house	N/A
LAAAHPAAL	In-house	N/A
LRTRFVYHL	In-house	N/A
VRFFFPSL	In-house	N/A
IYKTPPIKDF	In-house	N/A
CRVLCCYVL	In-house	N/A
LSEFCRVL	In-house	N/A
ARLSSPIVL	In-house	N/A
FYYVWSYV	In-house	N/A
LLYAHINAL	In-house	N/A
LTDEMIAQY	In-house	N/A
NYFNRMFHF	In-house	N/A
RYRPGTVAL	In-house	N/A
SYRSTYMIL	In-house	N/A
YFTSDYYQL	In-house	N/A
YRFRFRSVY	In-house	N/A
VMAPRTLIL	In-house	N/A

**Critical commercial assays**		

Isolate II genomic DNA kit	GC Biotech	Cat: BIO-52067
NebBuilder Hi-Fi Assembly	New England Biolabs	Cat: E5520

**Experimental models: Cell lines**		

A549	ATCC	Cat: CCL-185 RRID: CVCL_0023
HaCaT	Thermo Fisher	RRID:CVCL_0038
HeLa	ATCC	Cat: CCL-2 RRID:CVCL_0030
HEK293T	ATCC	Cat: CRL-3216; RRID:CVCL_0063
K562	Dr. B. Pang	RRID:CVCL_0004
MelJuSo	DSMZ	Cat: ACC 74 RRID: CVCL_1403
PC-3M	Urology department, LUMC	RRID: CVCL_9555
SiHa	Dr. M. Heemskerk	RRID: CVCL_0032
SKBR3	Medical Oncology department, LUMC	RRID:CVCL_B3MN
Raji	Dr. M. Heemskerk	RRID:CVCL_0511
U2OS	ATCC	Cat: UTB-96 RRID:CVCL_0042

**Oligonucleotides**		

Activating gRNA LILRB1 (AAGACTCAGAGATTTGTTCC)	Integrated DNA Technologies	N/A
Activating gRNA CD55 (GAGGTGCGGTCAGAGGGCTC)	Integrated DNA Technologies	N/A
Activating gRNA SDC1 (GCGTTCCGAAGGGGCCGGGA)	Integrated DNA Technologies	N/A
Activating gRNA SDC2 (AGAAGCAGGCTCAGGAGGGA)	Integrated DNA Technologies	N/A
Activating gRNA SDC4 (CGCAGGCCTCGCTTCCACTG)	Integrated DNA Technologies	N/A
KO gRNA CD55 (GGTACATCAATCTGACCATT)	Integrated DNA Technologies	N/A
KO gRNA GOLPH3 (GGAACGATTAGCTAAAAACC)	Integrated DNA Technologies	N/A
KO gRNA EXT1 (AAGTTACCAAAACATTCTAG)	Integrated DNA Technologies	N/A
KO gRNA EXT2 (TGGTTAAGCACATCGATGGA)	Integrated DNA Technologies	N/A
KO gRNA UGDH (GGTACATCAATCTGACCATT)	Integrated DNA Technologies	N/A
Genome-wide Calabrase activation library (sublibrary A+B)	Dr. J. Doench	Addgene cat: 1000000111
Genome-wide mouse CRISPR Brunello knockout library	Dr. D Root and Dr. J Doench	Addgene cat: 73178
Gibson assembly primer CD55 FW (ACACAGGACC GTTAATTAAGGTACCCACCATGACCGTCGCGC)	Integrated DNA Technologies	N/A
Gibson assembly primer CD55 RV (GGATCCTCTCG AGTGATATCTGAATTCAGTCAGCAAGCCCATGGT)	Integrated DNA Technologies	N/A
Gibson assembly primer EXT1 FW (ACACAGGACCG TTAATTAAGGTACCCACCATGCAGGCCAAAAAACGCT)	Integrated DNA Technologies	N/A
Gibson assembly primer EXT1 RV (GGATCCTCTCGAG TGATATCTGAATTCAAGTCGCTCAATGTCTCGGT)	Integrated DNA Technologies	N/A
Gibson assembly primer EXT2 FW (ACACAGGACCGT TAATTAAGGTACCCACCATGTGTGCGTCGGTCAAGT)	Integrated DNA Technologies	N/A
Gibson assembly primer EXT2 RV (GGATCCTCTCGAGT GATATCTGAATTCTAAGCTGCCAATGTTGGGG)	Integrated DNA Technologies	N/A
CD55 mutant primer Δ1 CD55_rv (GCCGTGTGGGGT CGTAGCTGCGAGGTGCCAAC)	Integrated DNA Technologies	N/A
CD55 mutant primer Δ2 CD55_rv (GCCGTGTGGGGT AAATCATGCCCTAATCCGGGAGAAATACG)	Integrated DNA Technologies	N/A
CD55 mutant primer Δ3 CD55_rv (GCCGTGTGGGGTG AAATTTATTGTCCAGCACCACCACAAATTG)	Integrated DNA Technologies	N/A
CD55 mutant primer Δ4 CD55_rv (GCCGTGTGGGGTAA ATCTCTAACTTCCAAGGTCCCACCAAC)	Integrated DNA Technologies	N/A
CD55 mutant primer CD55_muta_fw (ACCCCACACGGCCGGC)	Integrated DNA Technologies	N/A

**Recombinant DNA**		

pEGFP-C1	Clontech	Cat: 2487
CD55 IRES GFP Full Length (FL)	Addgene	Cat: 220874
Δ1 CD55 IRES GFP	Addgene	Cat: 220870
Δ2 CD55 IRES GFP	Addgene	Cat: 220871
Δ3 CD55 IRES GFP	Addgene	Cat: 220872
Δ4 CD55 IRES GFP	Addgene	Cat: 220873
Plasmid LentiCRISPRv2 puro	Addgene	Cat: 98290
Plasmid pMDLg/PRRE	Addgene	Cat: 12251
Plasmid pRSV-Rev	Addgene	Cat: 12253
Plasmid pCMV-VSV-G	Addgene	Cat: 8454
Plasmid dCas-VP64	Addgene	Cat: 61425
Plasmid pXPR_502	Addgene	Cat: 96923
Plasmid pLenti-P2A-Puro	Addgene	Cat: 211364

**Software and algorithms**		

BiaCore T200 Evaluation software	BIAevaluation Software	RRID: SCR_015936
FlowJo V.10	Treestar	RRID: SCR_008520; https://www.flowjo.com/solutions/flowjo
GraphPad Prism V.9	GraphPad	RRID: SCR_002798; http://www.graphpad.com/
NetMHC 4.0	DTU Health Tech	RRID: SCR_021651https://services.healthtech.dtu.dk/services/NetMHC-4.0/
PANDORA	Radboudumc Nijmegen	https://github.com/X-lab-3D/PANDORA
PinAPL-PY	University of San Diego, California	http://pinapl-py.ucsd.edu/
PyMol	PyMol	RRID:SCR_000305http://www.pymol.org/
Zen imaging software 2.1 V4	ZEISS	RRID:SCR_013672

**Other**		

DMEM	Gibco	Cat: 41966052
FCS	Bodinco	Cat: 5010
IMDM	Gibco	Cat: 12440
Series S Protein G sensor chip	Cytiva life sciences	Cat: 29179315
Protein G Sepharose 4 FF beads	Sigma-Aldrich	Cat: 4B200
RPMI-1640	Cytiva life sciences	Cat: SH30605

### Resource availability

#### Lead contact

Further information and requests for resources should be directed to and will be fulfilled up to reasonable request by the lead contact, Ferenc Scheeren (f.a.scheeren@lumc.nl).

#### Materials availability

This study generated four expression constructs for different CD55 truncation mutants that can be found on Addgene (full length, #220874, Δ1, #220870; Δ2, #220871; Δ3, #220872; Δ4, #220873). Peptides and tetramers were made in house.

#### Data and code availability

• All data reported in this paper will be shared by the lead contact upon request.
• This paper does not report original code.
• Any additional information required to reanalyse the data report in this paper is available from the lead contact upon reasonable request.

### Experimental model and study participant details

#### Cell lines

K562 (RRID:CVCL_0004) and Raji cells (RRID:CVCL_0511) were maintained in RPMI supplemented with 8% fetal calf serum (FCS). HEK293T (RRID:CVCL_0063), U2OS (RRID:CVCL_0042), A549 (RRID: CVCL_0023), HaCaT (RRID:CVCL_0038), HeLa (RRID:CVCL_0030), PC-3M (RRID: CVCL_9555), SiHa (RRID: CVCL_0032), and SKBR3 (RRID:CVCL_B3MN) cells were maintained in DMEM supplemented with 8% FCS. MelJuSo (RRID: CVCL_1403) cells were maintained in IMDM supplemented with 8% FCS. All cells were cultured at 37°C, and regularly checked for the absence of mycoplasma. The Raji, HEK293T, HeLa, U2OS and MelJuSo cell lines were authenticated by means of STR genotyping.

### Method details

#### Transfections, transductions

All transfections were performed using polyethyleneimine (Polysciences). To generate viral particles, HEK 293T cells were transfected with a lentiviral construct and three packaging plasmids: pCMV-VSV-G (Addgene #8454), pMDLg/pRRE (Addgene #12251) and pRSV-Rev (Addgene #12253). Supernatant containing viral particles was filtered and target cells were transduced using 8 μg/mL polybrene (EMD Millipore).

#### Tetramers and antibodies

Tetramers were generated in house as described in previous literature.^41^^,^^50^ All peptides used in this study were generated in house. Tetramer staining was performed for 30 min at 4°C in PBS 2% FBS at a 10nM concentration. Antibodies used in this study were anti-heparan sulfate chains (AMSBIO, F58-10E4), FITC anti-human CD55 (Biolegend, 311306), anti-human CD55 BRIC110 (ARP, 08-9402-2, targets SCR2 of CD55), anti-human CD55 BRIC216 (Biorad, MCA914T, targets SCR3 of CD55), anti-human CD55 MAB2009 (R&D systems, MAB2009-SP, targets SCR1 of CD55), anti-SDC2 APC (R&D systems, FAB2965A), anti-SDC4 APC (R&D systems, FAB29181A), anti-CD55 APC (Biolegend, #311311), or goat-anti-mouse APC (Biolegend, #405308).

#### Genome-wide CRISPR activation screening for recombinant HLA class-I tetramer binding

For CRISPR activation screening for transmembrane interactors of HLA, K562 cells were transduced with dCAS9-Blast (Addgene #61425), a kind gift from Feng Zhang, and the Calabrese genome-wide activation library (sublibrary A + B), kindly provided by John Doench (Addgene #1000000111). Transduced cells were then selected using blasticidin (5 μg/mL) and puromycin (2 μg/mL). After seven days, two batches of 50 million cells were stained with a pool of APC-labeled HLA-A -B and -C tetramers loaded with NLVPMVATV, TPRVTGGGAM and VRIGHLYIL peptides, respectively (each at 10nM). Cells that stained positive for tetramer binding (between 0.01 and 0.1%) were sorted using an Aria cell sorter and genomic DNA was isolated from the tetramer-positive and unsorted cells. After the sort, gDNA was isolated using the isolate II genomic DNA kit (GC Biotech) for both the unsorted and sorted populations and gDNA was amplified using the established protocol.^51^ Inserts were mapped to the reference and gRNA enrichment was analyzed using PinAPL-Py.^52^ For CRISPR activation screening for the interactors of HLA-C∗07:02-YRFRFRSVY, cells were prepared and analyzed as described earlier, but stained solely with PE-tagged HLA-C∗07:02 tetramers loaded with YRFRFRSVY. For both screens, sgRNA coverage and corresponding Gini coefficients were calculated using PinApl-Py analysis package as a library quality control.

#### Genome-wide CRISPR KO screen for HLA-C∗07:02-YRFR binding

For CRISPR KO screening of transmembrane interactors of HLA-C∗07:02-YRFR, MelJuSo cells were transduced with the Brunello genome-wide library, a gift from David Root and John Doench (Addgene #73178), containing 4 gRNAs per gene for a total of over 19,000 genes. Transduced cells were then selected using puromycin (1 μg/mL). After seven days, two batches of 30 million cells were stained with HLA-C∗07:02 tetramers loaded with YRFRFRSVY peptide coupled to PE. Cells that stained negative for HLA-C∗07:02-YRFR (between 0.01 and 0.1%) were sorted using an Aria cell sorter and gDNA was isolated from the tetramer-negative and unsorted cells. After the sort, gDNA was isolated using an isolate II genomic DNA kit (GC Biotech) for both the unsorted and sorted populations and gDNA was amplified using the established protocol.^51^ Inserts were mapped to the reference and analysis of gRNA enrichment was done using PinAPL-Py.^52^ sgRNA coverage of the screen and the corresponding Gini coefficient was calculated using the PinApl-Py analysis package.

#### Hit validation

For hit validation for the overexpression screens, individual gRNAs were cloned into the pXPR_502 vector (Addgene #96923) and K562 cells stably expressing dCas9 were transduced and selected using puromycin (2 μg/mL). Sequences for each guide are: CD55: 5′-GAGGTGCGGTCAGAGGGCTC-3′, LILRB1: 5′-AAGACTCAGAGATTTGTTCC-3', SDC1: 5′-GCGTTCCGAAGGGGCCGGGA-3′, SDC2: 5′-AGAAGCAGGCTCAGGAGGGA-3', SDC4: 5′-CGCAGGCCTCGCTTCCACTG-3'.

For hit validation for the KO screens, individual gRNAs were cloned into the LentiCRISPR V2 vector, a kind gift from Feng Zhang (Addgene # 52961), and MelJuSo cells were transduced and selected using puromycin (1 μg/mL). Sequences for each guide are: CD55: 5′- GGTACATCAATCTGACCATT-3′ GOLPH3: 5′-GGAACGATTAGCTAAAAACC-3′, EXT1: 5′-AAGTTACCAAAACATTCTAG-3’, EXT2: 5′-TGGTTAAGCACATCGATGGA-3′, UGDH: 5′-TAGACATGAATGACTACCAG-3’. Heparinase treatment was performed by incubation of cells in 10 μg/mL Heparinase II (R&D Systems, 6336-GH-010) at 37°C for 90 min. Cells were washed in PBS prior to staining and analysis. Pre-incubation with heparan sulfate (SelleckChem, S5992) 10 mg/mL.

#### Co-immunoprecipitations

To detect the HLA-C∗07:01-VRIG interaction with CD55 *in vitro*, 0.5 μg CD55-Fc (Sino Biological) was incubated at RT for 30 min with 0.5 μg HLA-C∗07:01-VRIG tetramer in NP-40 buffer (0.5% NP-40 (Merck Millipore), 150 mM NaCl, 5 mM MgCl_2_ and protease inhibitors (Roche)). Subsequently, CD55-Fc was immunoprecipitated using Protein G Sepharose 4 FF beads (Sigma-Aldrich). Beads were washed 4x with NP-40 buffer, after which SDS-sample buffer (2% SDS (BioSolve), 10% glycerol (Sigma-Aldrich), 5% β-mercaptoethanol (Sigma-Aldrich), 60 mM Tris-HCl (Combi Blocks) pH 6.8 and 0.01% bromophenol blue (Merck)) was added to the beads. Samples were boiled before loading and proteins were separated by SDS-PAGE and transferred to Nitrocellulose filters (Cytiva). Blocking of the filter and antibody incubations was done in PBS supplemented with 0.1% (V/V) Tween20 (Sigma-Aldrich) and 5% (W/V) BSA (Santa Cruz). Blots were stained using streptavidin 800 (LI-COR) and anti-human Fc 680 (LI-COR) and imaged using the Odyssey Imaging System (LI-COR).

#### Microscopy

HeLa WT and HeLa CD55 KO cells were fixed with 4% paraformaldehyde, cytospun and blocked for 30 min at room temperature with Superblock (Thermo Fisher). Cells were stained with HLA-C∗07:01-VRIG tetramer PE or APC anti-human CD55 (Biolegend, 311311) for 5 h at room temperature followed by a DAPI staining for 5 min at room temperature. Cells were mounted with ProLong Gold (Thermo Fisher) and slides were visualized on a LSM 700 confocal microscope and analyzed using Zen imaging software 2.1 V4.

#### Flow cytometry

Flow cytometry experiments were conducted on an Aurora 3L (Cytek Biosciences) or LSR-II flow cytometer (BD Biosciences) and data was analyzed using FlowJo (V10.8). Cell sorting was performed on a BD FACSARIA III (BD Biosciences).

#### CD55 truncation analysis and rescue experiments

CD55 was subcloned between the XhoI and BamHI sites of the C1-IRES-GFP vector of C1-GFP/RFP vector system (Clontech). CD55 truncation plasmids were generated from the CD55-IRES-GFP expression plasmid using IVA mutagenesis cloning.^53^ To delete SCR domains, the signal sequence from amino acid 1–34 was kept in all mutants. Primers, deleted regions and plasmid details can be found in Table S2. HEK 293T cells were transfected 1 day prior to analysis with a plasmid containing a CD55 truncation mutant fused to GFP via an IRES sequence. GFP+ cells were gated prior to analysis. Overexpression plasmids for rescue experiments were generated by subcloning cDNA of CD55, EXT1, or EXT2 (ORFeome library, Horizon Discovery, Cat: OHS5221) into a pLenti-P2A-Puro vector (Addgene #211364) with Gibson assembly using a NebBuilder Hi-Fi assembly kit (New England Biolabs) with the following primers: CD55 FW 5′ ACACAGGACCGTTAATTAAGGTACCCACCATGACCGTCGCGC 3′, CD55 RV 5′ GGATCCTCTCGAGTGATATCTGAATTCAGTCAGCAAGCCCATGGT 3′, EXT1 FW 5′ ACACAGGACCGTTAATTAAGGTACCCACCATGCAGGCCAAAAAACGCT 3′, EXT1 RV 5′ GGATCCTCTCGAGTGATATCTGAATTC`AAGTCGCTCAATGTCTCGGT 3′, EXT2 FW 5′ ACACAGGACCGTTAATTAAGGTACCCACCATGTGTGCGTCGGTCAAGT 3′, EXT2 RV 5′ GGATCCTCTCGAGTGATATCTGAATTCTAAGCTGCCAATGTTGGGG 3’. HeLa CD55 KO cells were transfected with pLenti-CD55 2 days prior to analysis and selected using puromycin (1 μg/mL). MelJuSo EXT1 KO or EXT2 KO cells were transfected with pLenti-EXT1 or pLenti-EXT2 2 days prior to analysis and selected using puromycin (1 μg/mL).

#### Surface plasmon resonance

SPR assays were performed on a Biacore T200 (GE Healthcare) at 25°C. CD55-Fc (Sino Biological) was immobilized on a Series S Protein G sensor chip (Cytiva) on one flow cell, while a blank flow cell was used as reference. A concentration series of HLA-C∗07:01-VRIG tetramers or monomers was tested for binding in running buffer (150mM NaCl, 0.05% BSA, 0.05% v/v Tween-20, pH 7,5) on both flow cells. Reference-subtracted curves were analyzed using BiaCore T200 Evaluation software to determine *k*_on_ and *k*_off_ values, which were consecutively used to calculate the interaction value (*K*_D_). Curves and steady-state equilibrium binding values were plotted in GraphPad Prism 9.

#### Modelling

Peptide-HLA class-I structures were modeled using PANDORA v1.0.0 using their allotype and peptide as input.^54^ Visualization and surface binding area estimation were performed in PyMol.

### Quantification and statistical analysis

All screening experiments were performed with two biological replicates, other experiments were performed at least three times unless otherwise indicated. Data are represented as mean ± SD unless indicated otherwise.

## Supplemental information

Document S1. Figures S1–S4, Tables S2 and S3

Table S1. Raw data from CRISPR-Cas9 activation and knockout screens for HLA class-I tetramer binding, related to Figure 1 and Figure 3

Table S4. HLA tetramer sequences of peptides in different HLA tetramer allotypes that were tested for binding to HeLa cells, related to Figure 3
