# Supplementary material for: CRISPR-Cas9 screening reveals a distinct class of MHC-I binders with precise HLA-peptide recognition
Source: iScience. 2024 May 27;27(6):110120. doi: 10.1016/j.isci.2024.110120 (PMC11209011; doi:10.1016/j.isci.2024.110120)
Supplement: Document S1. Figures S1–S4, Tables S2 and S3 [file mmc1.pdf]

iScience, Volume *27*

Supplemental information

CRISPR-Cas9 screening reveals a distinct class of MHC-I binders with precise HLA-peptide recognition

Tom A.W. Schoufour, Anneloes van der Plas - van Duijn, Ian Derksen, Marije Melgers, Jacqueline M.F. van Veenendaal, Claire Lensen, Mirjam H.M. Heemskerk, Jacques Neefjes, Ruud H.M. Wijdeven, and Ferenc A. Scheeren

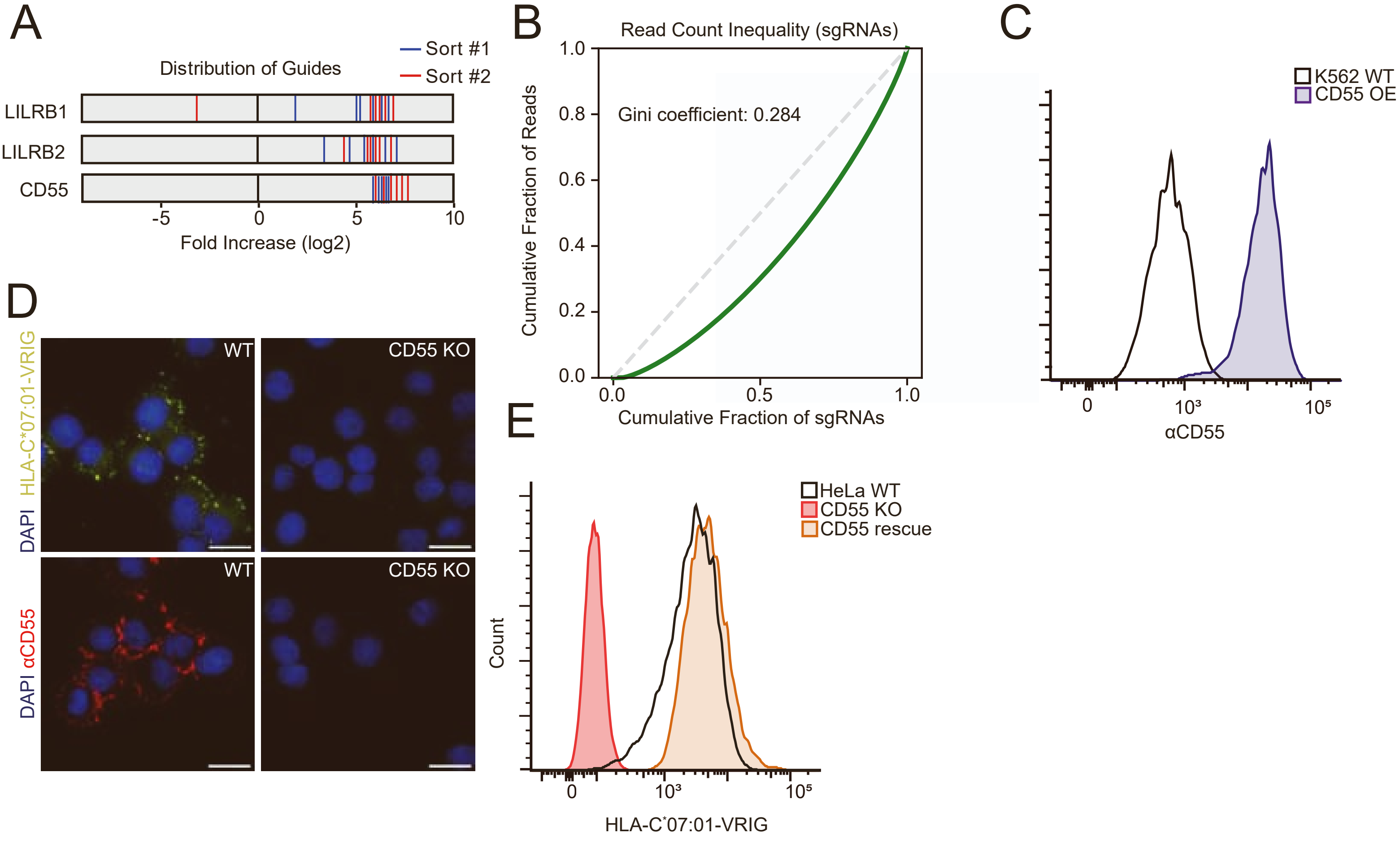

Figure S1. Receptor-ligand CRISPR/Cas9 activation screen reveals that CD55 interacts with HLA-C*07:01-VRIG tetramers, supplemental to Figure 1. (A) gRNA enrichment for top hits of the screen, red and blue stripes represent relative enrichment of individual gRNAs of two replicate sorts compared to unsorted cells. (B) sgRNA coverage as found in the control population of the activation screen with the corresponding Gini coefficient (C) K562 cells stably expressing dCas9 and transduced with a gRNA targeting either CD55 or a control guide were analyzed for surface expression of CD55 by flow cytometry. (D) Confocal microsco-py images of HeLa control or CD55 KO cells stained with HLA-C tetramer (yellow) or anti-CD55 (red) and DAPI (blue). Scale bar represents 20µm. (E) HeLa WT or HeLa CD55 KO cells were transiently transfected with a CD55 OE plasmid, stained with HLA-C*07:01-VRIG tetramers and analyzed by flow cytometry. WT, wildtype; OE, overexpression; KO, knockout; CD55 rescue, CD55 knockout cells transduced with a CD55 overexpression plasmid.

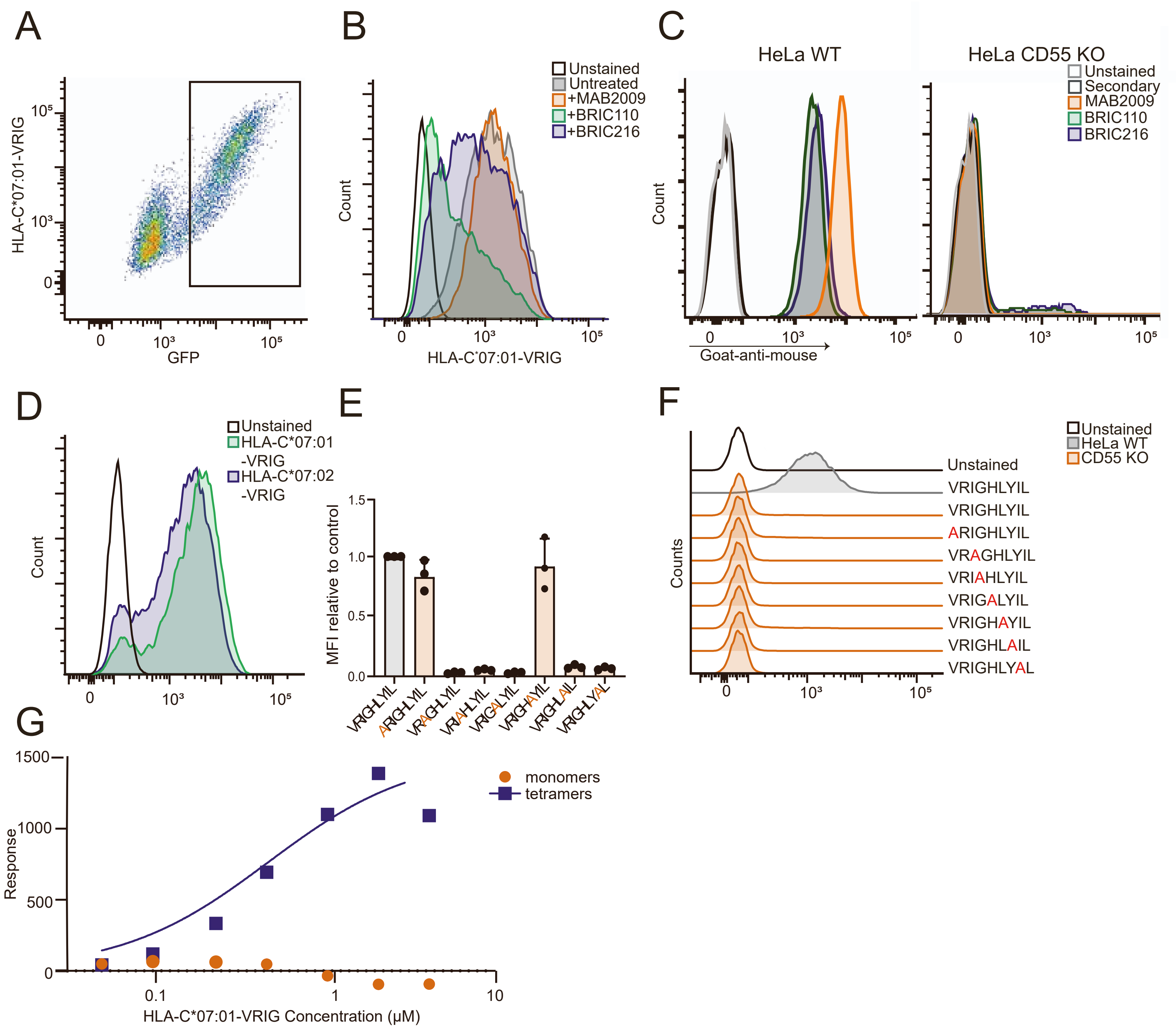

Figure S2. Interaction of CD55 with HLA-C*07:01-VRIG is allotype and peptide specific, supplemental to Figure 2. (A) Gating strategy used for analysis of HLA-C*07:01 tetramer staining on HEK293T cells. Example shows cells that were transfected with a plasmid containing GFP and CD55. GFP+ positive cells were analyzed for staining with HLA-C*07:01-VRIG. (B) PC-3M cells were stained with HLA-C*07:01-VRIG tetramers after pre-incubation with blocking antibodies targeting different SCR domains on CD55 and analyzed by flow cytometry. (C) HeLa wildtype and CD55 KO cells were stained with all CD55 blocking antibodies followed by secondary goat-anti-mouse antibodies, and analyzed by flow cytometry. (D) K562 LILBR1 overexpressing cells were stained with either HLA-C*07:01 or HLA-C*07:02 tetram ers loaded with the VRIG peptide and analyzed by flow cytometry. (E) Relative staining of respective alanine mutants of HLA-C*07:01-VRIG to the original HLA-C*07:01-VRIG tetramer on HeLa as analyzed by flow cytometry. Data are repre sented as mean ± SD. (F) HeLa WT or CD55 KO cells were stained with HLA-C*07:01 tetramers loaded with different alanine mutants of the VRIGHLYIL peptide and analyzed by flow cytometry. (G) CD55-Fc was immobilized on a Prot-G chip for SPR data using HLA-C*07:01-VRIG monomers (orange) or tetramers (purple) as analytes. Data of the tetramers was fitted to one site, specific binding. SCR, short consensus repeat; KO, knockout; MAB2009 BRIC110 and BRIC216 are all CD55 binding antibodies.

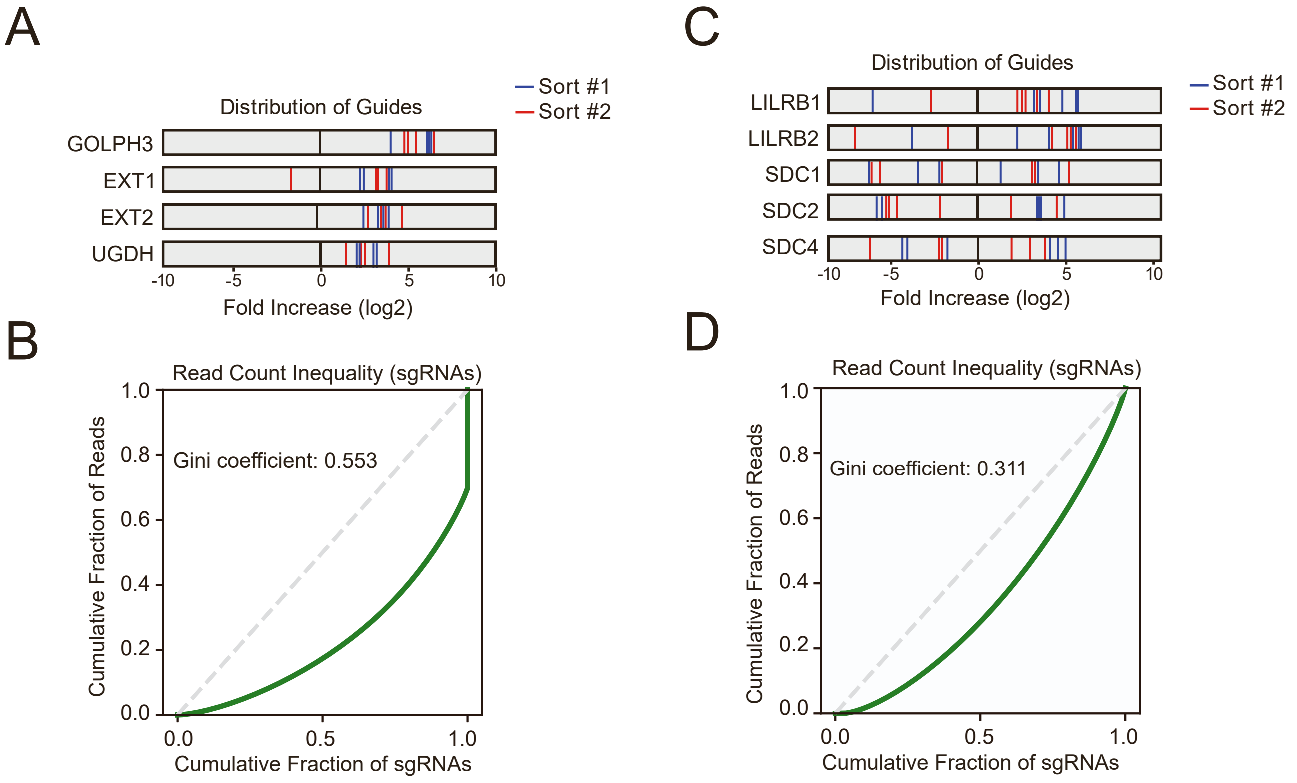

Figure S3. CRISPR/Cas9 activation and KO screens identify an interaction between HLA-C*07:02-YRFR and heparan sulfate chains, supplemental to Figure 3. (A) gRNA enrichment for top hits of the KO screen, red and blue stripes represent enrichment of individual gRNAs of two replicate sorts compared to unsorted cells. (B) sgRNA coverage as found in the control population of the KO screen with the corresponding Gini coefficient (C) gRNA enrichment for top hits of the activation screen, red and blue stripes represent enrichment of individual gRNAs of two replicate sorts compared to unsorted cells. (D) sgRNA coverage as found in the control population of the activation screen with the corresponding Gini coefficient.

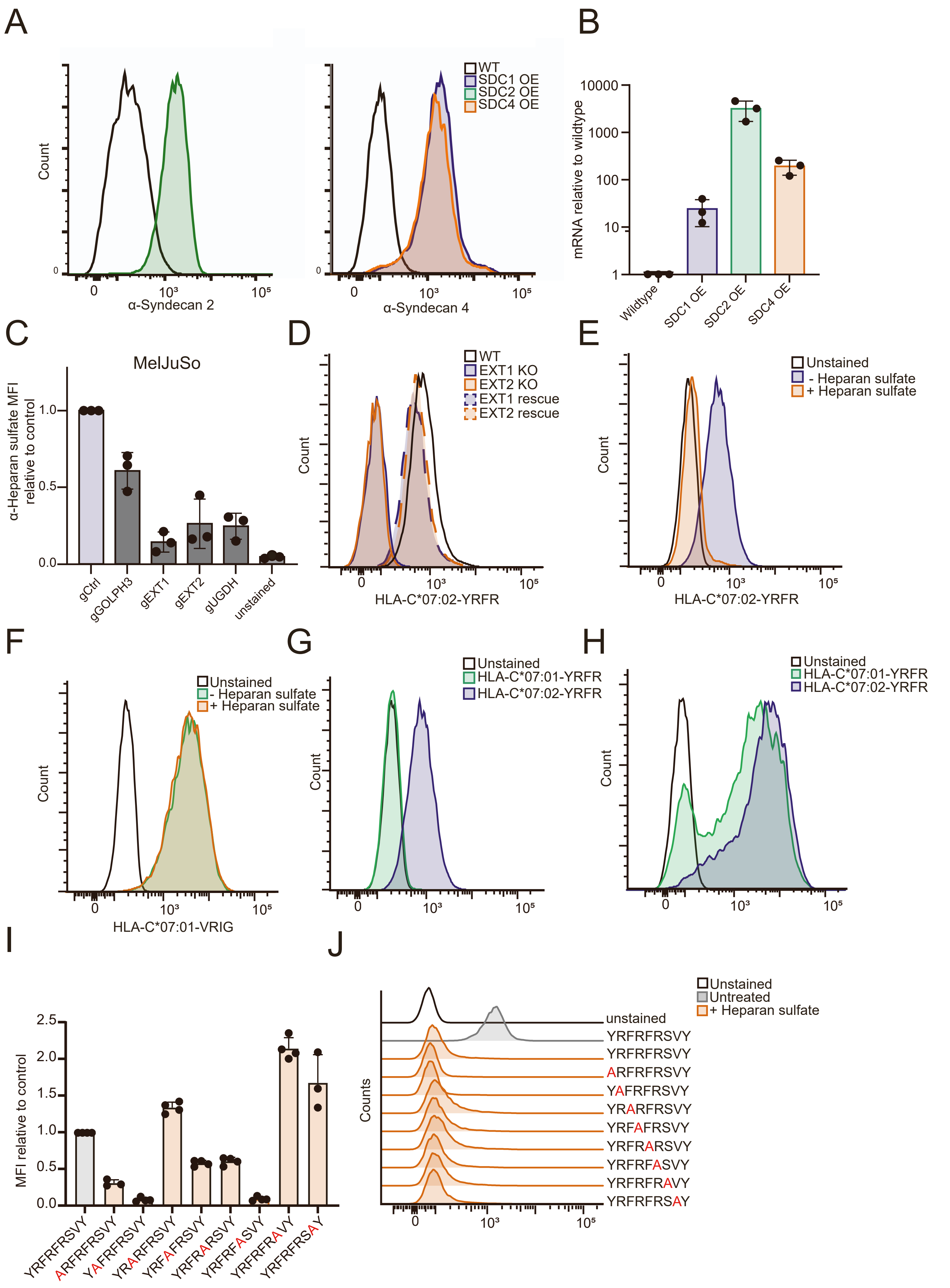
A
Count
α-Syndecan 2
α-Syndecan 4
WT
SDC1 OE
SDC2 OE
SDC4 OE
B
mRNA relative to wildtype
Wildtype
SDC1 OE
SDC2 OE
SDC4 OE
C
MelJuSo
α-Heparan sulfate MFI relative to control
gCtrl
gGOLPH3
gEXT1
gEXT2
gUGDH
unstained
D
WT
EXT1 KO
EXT2 KO
EXT1 rescue
EXT2 rescue
HLA-C*07:02-YRFR
E
Unstained
- Heparan sulfate
+ Heparan sulfate
HLA-C*07:02-YRFR
F
HLA-C*07:01-VRIG
G
HLA-C*07:01-YRFR
HLA-C*07:02-YRFR
H
I
MFI relative to control
YRFRFRSVY
ARFRFRSVY
YAFRFRSVY
YRARFRSVY
YRFAFRSVY
YRFRARSVY
YRFRFASVY
YRFRFRAVY
YRFRFRSAY
J
Untreated
Counts
unstained

Figure S4. Interaction between HLA-C*07:02-YRFR and heparan sulfate chain is allotype and peptide specific, supplemental to Figure 4. (A) K562 cells overexpressing SDC1, -2 or -4 were stained with either anti-SDC2 or -4 antibodies, and analyzed by flow cytometry. (B) mRNA levels for SDC1, -2 and -4 normalized to mRNA levels of control K562 cells. (C) MelJuSo cells were transduced with a gRNA knocking out GOLPH3, EXT1, EXT2 or UGDH, stained for surface heparan sulfate and analyzed by flow cytometry. (D) MelJuSo EXT1 or EXT2 KO cells were transiently transfected with an overexpression plasmid for EXT1 or EXT2, stained with HLA-C*07:02 tetramers and analyzed by flow cytometry. (E) HeLa cells were stained with HLA-C*07:02-YRFR tetramers pre-incubated with recombinant heparan sulfate chains when indicated and analyzed by flow cytometry. (F) HeLa cells were stained with HLA-C*07:01-VRIG tetramers pre-incubated with recombinant heparan sulfate chains when indicated and analyzed by flow cytometry. (G) HeLa cells were stained with either HLA-C*07:01 or HLA-C*07:02 tetramers loaded with YRFR peptide and analyzed by flow cytometry (H) K562 LILRB1 overexpressing cells were stained with either HLA-C*07:01 or HLA-C*07:02 tetramers loaded with YRFR peptide and analyzed by flow cytometry. (I) MelJuSo cells were stained with HLA-C*07:02 tetramers loaded with different alanine mutants of the YRFRFRSVY peptide and analyzed by flow cytometry. (J) MelJuSo cells were pre-incubated with recombinant heparan sulfate chains when indicated and stained with HLA-C*07:02 tetramers loaded with different alanine mutants of the YRFRFRSVY peptide and analyzed by flow cytometry. Data represent three independent experiments, and bar graphs represent the mean ± SD. SDC, syndecan; WT, wildtype; OE, overexpression; KO, knockout.

Table S2, related to Figure 2. List of primers used for the generation of CD55 truncation plasmids. Each generated plasmid has the given amino acids deleted and are deposited to addgene.

Primer	Reverse primer used	Region deleted
**Δ1 CD55_rv**	GCCGTGTGGGGTCGTAGCTGCGAGGTGCCAAC	35-96
**Δ2 CD55_rv**	GCCGTGTGGGGTAAATCATGCCCTAATCCGGGAGAAATACG	35-161
**Δ3 CD55_rv**	GCCGTGTGGGGTGAAATTTATTGTCCAGCACCACCACAAATTG	35-222
**Δ4 CD55_rv**	GCCGTGTGGGGTAAATCTCTAACTTCCAAGGTCCCACCAAC	35-385
**CD55_muta_fw**	ACCCCACACGGCCGGC	

Table S3, related to Figure 2 and 4. Affinity of peptides in applicable HLA-C*07 tetramers as determined by NetMHC 4.0 https://services.healthtech.dtu.dk/services/NetMHC-4.0/. NA is not applicable.

Peptide	Affinity in HLA-C*07:01 [nM]	Affinity in HLA-C*07:02 [nM]
**VRIGHLYIL**	133.8	647
**ARIGHLYIL**	169.2	NA
**VRAGHLYIL**	122.3	NA
**VRIAHLYIL**	102.0	NA
**VRIGALYIL**	551.1	NA
**VRIGHAYIL**	165.2	NA
**VRIGHLAIL**	252.6	NA
**VRIGHLYAL**	211.0	NA
**YRFRFRSVY**	22.5	36.8
**ARFRFRSVY**	NA	376.0
**YAFRFRSVY**	NA	693.1
**YRARFRSVY**	NA	117.5
**YRFAFRSVY**	NA	43.3
**YRFRARSVY**	NA	72.9
**YRFRFASVY**	NA	33.7
**YRFRFRAVY**	NA	34.8
**YRFRFRSAY**	NA	28.3
